# A Fragment of 21 ORFs Around the Direct Repeat (DR) Region of  *Mycobacterium tuberculosis*  is Absent From the Other  Sequenced Mycobacterial Genomes: Implications for the Evolution of the DR Region

**DOI:** 10.1002/cfg.380

**Published:** 2004-03

**Authors:** Karina Caimi, Angel Cataldi

**Affiliations:** Instituto de Biotecnología CICVyA/INTA, Los Reseros y Las Cabañas Castelar 1712 Argentina

## Abstract

The direct repeat (DR) region is a singular locus of the *Mycobacterium tuberculosis* complex genome. This region consists of 36 bp repetitive sequences separated by non-repetitive unique spacer sequences. Around this region there are several genes coding for proteins of unknown function. To determine whether the *M. smegmatis, M. avium, M. marinum* and *M. leprae* genomes contain sequences and ORFs similar to those of the DR locus of the *M. tuberculosis* complex, we analysed the corresponding regions in these species. As a first step, some conserved genes that flank the DR genes [*Rv2785c (rpsO), Rv2786c (ribF), Rv2790c (ltp1 ), Rv2793c (truB), Rv2800, Rv2825, Rv2828, Rv2831 (echA16 ), Rv2838 (rbfA)* and *Rv2845 (proS )*] were used as markers to locate the corresponding orthologues in *M. smegmatis, M. avium, M. marinum* and *M. leprae in silico*. Most of these *M. tuberculosis* marker genes have highly similar orthologues located in the same order and orientation in the other mycobacteria. In contrast, no orthologues were found for ORFs *Rv2801–Rv2824*, suggesting that these genes are unique to *M. tuberculosis* within the genus *Mycobacterium*.We observed that in *M. smegmatis* and *M. avium, Rv2800* and *Rv2825* are adjacent. This observation was experimentally confirmed by PCR. In conclusion, as the DR locus and the ORFs around it are absent in *M. smegmatis* and *M. avium* and, as it is possible that these species are older than *M. tuberculosis*, we postulated that the DR locus was acquired by the *M. tuberculosis* complex species or by an ancestor bacterium.


**Comparative and Functional Genomics**
*Comp Funct Genom* 2004; **5**: 116–122.
Published online in Wiley InterScience (www.interscience.wiley.com). **DOI:** 10.1002/cfg.380

**Short Communication**

### A fragment of 21 ORFs around the direct repeat (DR) region of *Mycobacterium tuberculosis* is absent from the other sequenced mycobacterial genomes: implications for the evolution of the DR region

Karina Caimi and Angel Cataldi*
*Institute of Biotechnology, CICVyA-INTA Castelar, Argentina*

**Correspondence to:
Angel Cataldi, Instituto de Biotecnología, CICVyA/INTA, Los Reseros y Las Cabañas, 1712 Castelar, Argentina.
E-mail: acataldi@cnia.inta.gov.ar*

### Abstract

**The direct repeat (DR) region is a singular locus of the *Mycobacterium tuberculosis* complex genome. This region consists of 36 bp repetitive sequences separated by non-repetitive unique spacer sequences. Around this region there are several genes coding for proteins of unknown function. To determine whether the *M. smegmatis*, *M. avium*, *M. marinum* and *M. leprae* genomes contain sequences and ORFs similar to those of the DR locus of the *M. tuberculosis* complex, we analysed the corresponding regions in these species. As a first step, some conserved genes that flank the DR genes [*Rv2785c* (*rpsO*), *Rv2786c* (*ribF*), *Rv2790c* (*ltp1*), *Rv2793c* (*truB*), *Rv2800*, *Rv2825*, *Rv2828*, *Rv2831* (*echA16*), *Rv2838* (*rbfA*) and *Rv2845* (*proS*)] were used as markers to locate the corresponding orthologues in *M. smegmatis*, *M. avium*, *M. marinum* and *M. leprae in silico*. Most of these *M. tuberculosis* marker genes have highly similar orthologues located in the same order and orientation in the other mycobacteria. In contrast, no orthologues were found for ORFs *Rv2801–Rv2824*, suggesting that these genes are unique to *M. tuberculosis* within the genus *Mycobacterium*. We observed that in *M. smegmatis* and *M. avium, Rv2800* and *Rv2825* are adjacent. This observation was experimentally confirmed by PCR. In conclusion, as the DR locus and the ORFs around it are absent in *M. smegmatis* and *M. avium* and, as it is possible that these species are older than *M. tuberculosis*, we postulated that the DR locus was acquired by the *M. tuberculosis* complex species or by an ancestor bacterium.** **Copyright © 2004 John Wiley & Sons, Ltd.**

**Keywords:** *Mycobacterium*; **DR region; insertion/deletion; tuberculosis**

Received: 24 July 2003
Revised: 15 December 2003
Accepted: 22 December 2003

### Introduction

The direct repeat (DR) region of the *Mycobacterium tuberculosis* complex consists of 36 bp repetitive sequences separated by non-repetitive unique spacer sequences whose lengths vary from 27 to 41 bp (Hermans *et al.*, 1991; Kamerbeek *et al.*, 1997; van Embden *et al.*, 2000; Caimi *et al.*, 2001). *M. tuberculosis* complex isolates are polymorphic (Fang *et al.*, 1998; Groenen *et al.*, 1993) at the DR region because they can differ in the presence or absence of the different spacers and adjacent DRs. In spoligotyping, a technique to distinguish isolates, the DR region is amplified by PCR using DR-specific biotinylated primers. The presence or absence of each of the spacers is determined by hybridizing the amplified DNA to a membrane containing oligonucleotides corresponding to each of these spacer sequences (Kamerbeek *et al.*, 1997; Sola *et al.*, 1999; van Soolingen *et al.*, 1993).

Copyright © 2004 John Wiley & Sons, Ltd.

The function of the DR locus in *M. tuberculosis* is presently unknown, but its omnipresence in the *M. tuberculosis* complex and the conservation in many different isolates of the sequence and order of the DRs and spacers suggest that the region could have a biological function. The DR region in *M. tuberculosis* is a hot spot of integration of the IS*6110* insertion element (Fang *et al.*, 1998; Hermans *et al.*, 1991). The polymorphisms in the DR associated with IS*6110* insertions have been described in different strains, including some outbreak isolates (Fang *et al.*, 1998; Groenen *et al.*, 1993). The different species of the *M. tuberculosis* complex (*M. tuberculosis*, *M. bovis*, *M. africanum*, *M. microti* and *M. canettii*) show distinctive characteristics, such as the presence and absence of particular spacers (Caimi *et al.*, 2001; van der Zanden *et al.*, 2002). Interestingly, there is low homology between the spacers (with the exception of spacers 40 and 85), and they contain a huge amount of stop codons (unpublished observations), suggesting that they do not derive from a gene interrupted by the insertions of the 36 bp direct repeats. Notably, a unique set of spacers is present in *M. canettii*. No significant homology has been reported between the *M. tuberculosis* DR sequence and DNA from any other bacteria. After the recent sequencing of various bacterial genomes, it has been postulated that the DR sequence of *M. tuberculosis* belongs to the clustered regularly interspaced short palindromic repeats (CRISPR) family of repetitive DNA (Jansen *et al.*, 2002). In spite of this important advance in the understanding of the ubiquity and structure of CRISPR-type sequences, their biological function, if any, remains unclear (Mojica *et al.*, 1995). In consequence, the DR region remains as an enigma of the *M. tuberculosis* complex genome.

In this work we have analysed the genes surrounding the *M. tuberculosis* DR region in the *M. smegmatis, M. avium, M. marinum* and *M. leprae* genomes. The goal was to determine whether the equivalent regions have a similar ORF organization and to find any resemblances to the DR sequences.

### Material and methods

### BLAST searches

Amino acid sequence alignments were made by searching public databases via the Internet. For *M. smegmatis* and *M. avium* the BLASTp search facility at the TIGR Unfinished Microbial Genomes site (**http://www.tigr.org/tdb/mdb/mdbinprogress.html**) was used. The parameters used were: program, BLASTp; alignments, 5; matrix, blosum62; cut-off, none; strand, default expect, 0.1; description, 5; and filter, none. At the Pasteur Institute sites, BLASTp searches were performed for *M. leprae* (**http://genolist.pasteur.fr/Leproma/**) using the NCBI BLAST version 2 algorithm, and for *M. ulcerans* (**http://genopole.pasteur.fr/Mulc/BuruList.html**) using the parameters: −F, F; −e, 1e−3; −q, −1; −b, 20; −v, 20; and −S, 3. Finally, TBLASTn searches were performed at the Sanger Centre sites for *M. marinum* (**http://www.sanger.ac.uk/Projects/M_marinum/**) and *S. coelicolor* (**http://www.sanger.ac.uk/Projects/S_coelicolor/**) selecting genome sequence EMBL entries.

The Artemis software (release 4 for Macintosh, Sanger Center) was used to annotate the selected regions of *M. smegmatis*, *M. avium*, *M. leprae* and *M. marinum*.

BLASTp searches of ORFs identified by Artemis were performed using the NCBI services (**http://www.ncbi.nlm.nih.gov/BLAST**) against the nr database.

### PCR

Amplifications were performed using Taq-DNA polymerase (Promega) under standard conditions, in a volume of 50 µl. Briefly, the concentration of dNTPs was 0.2 mM each, and 20 pmol of each primer were used. Cycling conditions were: one cycle of 94 °C for 3 min, followed by 35 cycles of: 94 °C for 1 min, 56 °C for 1 min, 1 min at 72 °C; followed by 10 min at 72 °C. A total of 2 ng of genomic DNA was used as template. The *M. avium* genome was amplified with the primers: Maup2DR (CGGCGGTTACCGCGACGATAC), which anneals to Rv2800-like gene, and MalowDR (GCGATTGGCGGGAGCGAAATG), which anneals to Rv2825-like gene. *M. smegmatis* was amplified with the primers MsupDR (TCCCCTGCCCGCCGATCTCTA), which anneals to Rv2800-like gene, and MslowDR (CGACTACGGAGGCTGCAAGAG), which anneals to Rv2825-like gene.

Copyright © 2004 John Wiley & Sons, Ltd.

### Results

As a first step, we identified the following genes that are described as conserved in the Tuberculist database and located around the DR region in the *M. tuberculosis* genome: *Rv2785c* (*rpsO*), *Rv2786c* (*ribF*), *Rv2790c* (*ltp1*), *Rv2793c* (*truB*), *Rv2800*, *Rv2825*, *Rv2828*, *Rv2831* (*echA16*), *Rv2838* (*rbfA*) and *Rv2845* (*proS*) (Figure 1). As highly conserved counterparts of all these genes are present in several other bacterial species, they were used as markers to locate, *in silico*, the corresponding orthologues in the *M. smegmatis*, *M. avium*, *M. leprae* and *M. marinum* genomes, and thus identify the regions where DR-like sequences might be found. In *M. tuberculosis*, the DR region is located between *Rv2800* and *Rv2825*.

BLASTp searches against four mycobacterial genomes (*M. smegmatis*, *M. avium*, *M. leprae* and *M. marinum*) indicate that in these bacteria most of the *M. tuberculosis* marker genes have highly similar orthologues, which are also found in the same order and orientation. Table 1 shows the data from *M. tuberculosis*, *M. smegmatis* and *M. avium*. Importantly, there are no orthologues for ORFs *Rv2801–Rv2824* in either *M. smegmatis* or *M. avium*, suggesting that these genes are unique to *M. tuberculosis* within the genus *Mycobacterium*, at least in those mycobacteria whose genomes were sequenced. The absence of these ORFs is reflected by the distance between the orthologues of *Rv2800* and *Rv2825*, which is 24.5 kb in *M. tuberculosis*; but only 2.2 kb in *M. avium* and 2.2 kb in *M. smegmatis*. Similar observations were made in *M. leprae* and *M. marinum* (Table 2). No repetitive sequences similar to the DR of *M. tuberculosis* were found.

As the *M. avium* genome is not yet annotated, the region between the orthologues of *truB* and *proS* was located by text phrase search in the whole genome text file provided by TIGR. This region, comprising 20 527 bp, was selected, cut from the text file, and its ORFs analysed using Artemis. In turn, the *M. smegmatis* region between *truB* and *rbfA* was located in the same way on contig 3315 provided by TIGR, from which 19 002 bp were cut and analysed with Artemis. Every ORF present in these regions was BLAST-searched against the Tuberculist database. We observed that orthologues of *Rv2801–Rv2824* are not present in *M. smegmatis* and *M. avium*, and that *Rv2800* and *Rv2825* are adjacent in these genomes (Figure 1). Thus, a 24.5 kb segment of the *M. tuberculosis* genome that contains

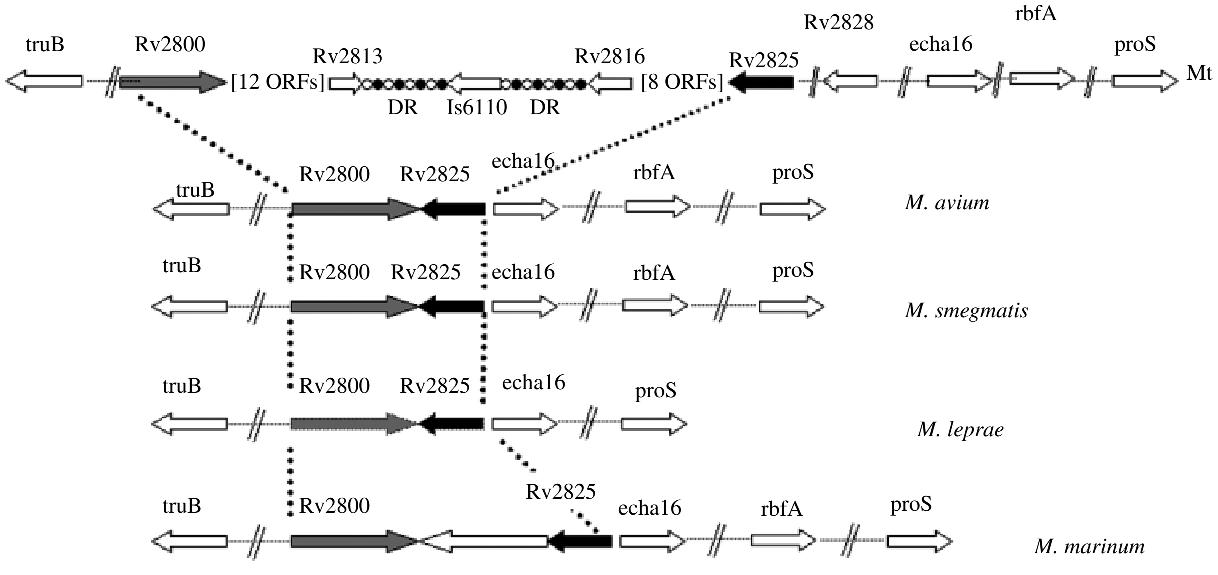

**Figure 1.** Schematic representation of ORFs in the DR region and surrounding area in *M. tuberculosis* and other mycobacteria. Mt, *M. tuberculosis*. Arrow blocks represent ORFs. The names of genes are given above the ORFs (as they appear in Tuberculist). Circles represent the DR region. To facilitate comparison, *Rv2800* and *Rv2825* are coloured grey and black, respectively

Copyright © 2004 John Wiley & Sons, Ltd. 

**Table 1.** Amino acid similarities and genomic positions of genes in, or close to, the DR region in *M. smegmatis and M. avium*

| *M. tuberculosis* gene | *M. smegmatis* | | *M. avium* | |
|---|---|---|---|---|
| | E value | Position[a] | E value | Position[b] |
| Rv2785c/rpsO | 2.3e−38 | 150 629 | 3.0e−31 | 3 805 416 |
| Rv2786c/ribF | 1.1e−153 | 151 723 | 4.8e−161 | 3 806 537 |
| Rv2790c/ltp1 | 7.9e−50[1] | — | 5.9e−189 | 3 809 914 |
| Rv2793c/truB | 5.7e−106 | 155 877 | 4.0e−108 | 3 810 970 |
| Rv2800 | 7.7e−182 | 159 842 | 1.9e−205 | 3 813 339 |
| Rv2801 | 2.6e−7[1] | — | 0.27 | — |
| Rv2802 | None | — | None | — |
| Rv2803 | 0.31 | — | None | — |
| Rv2804c | 0.00045 | — | 1 | — |
| Rv2805 | None | — | 0.51 | — |
| Rv2806 | 0.37 | — | 2.7e−14[4] | — |
| Rv2807 | None | — | None | — |
| Rv2808 | None | — | 0.018 | — |
| Rv2809 | 0.31 | — | 0.57 | — |
| Rv2810c | 1.1e−18[2] | — | None | — |
| Rv2811 | 0.030 | — | 0.84 | — |
| Rv2812 | 1.0e−07[2] | — | None | — |
| Rv2813 | None | — | 3 | — |
| Rv2817c | None | — | None | — |
| Rv2818c | None | — | None | — |
| Rv2819c | None | — | None | — |
| Rv2820c | 0.59 | — | None | — |
| Rv2821c | None | | None | — |
| Rv2822 | None | | 0.99 | |
| Rv2823c | 0.61 | | 9.3 | |
| Rv2824c | None | | None | |
| Rv2825c | 5.2e−59[3] | 162 020 | 1.1e−72 | 3 815 567 |
| Rv2827 | 0.21 | — | 0.85 | — |
| Rv2828 | 1.7e−67[3] | 162 020 | 5.3e−73 | 3 815 567 |
| Rv2829 | 0.15 | — | 0.14 | — |
| Rv2830 | 0.2 | — | 0.24 | — |
| Rv2831/echA16 | 2.4e−87 | 162 357 | 1.2e−110 | 3 815 922 |
| Rv2836c/dinF | 1.3e−171 | | 0.58 | — |
| Rv2838c/rbfA | 2.0e−36 | 174 879 | 7.2e−42 | 3 818 714 |
| Rv2845c/proS | 5.8e−257 | ND | 5.8e−257 | 3 826 596 |

[a] Position on contig 3315.
[b] Position on genome.
[1] Contig 3310;
[2] contig 3311;
[3] note that Rv2825 (215 aa) and Rv2828c are highly similar (181 aa);
[4] located at position 2 262 112 on the *M. avium* genome.

*Rv2801–Rv2824* is completely absent from the *M. smegmatis* and *M. avium* genomes.

By examining in detail the putative *Rv2800* and *Rv2825* orthologues in *M. avium*, it was observed that these genes overlap at their 3′ ends by 41 bp in this species. In the *M. smegmatis* genome these genes are separated by 23 bp. We also noted that the *ugp* operon is located elsewhere in the *M. avium* genome and that the *dinF* gene is absent from the *M. avium* genome (data not shown). The *M. leprae* genome is annotated, so it was easier to analyse the genetic structure of this region. The orthologues of Rv2800 and Rv2825 are ML1550 and ML1551, respectively. Both are pseudogenes, and they are adjacent and divergently transcribed, as in the other species. In *M. marinum*, there is an ORF between the Rv2800- and Rv2825-like genes (Figure 1). This ORF has limited similarity to the *otsB1* probable trehalose-6-phosphatase and is highly similar to a hypothetical protein from *Streptomyces lavendulae* (data not shown). In *Streptomyces coelicolor* there are no orthologues of the Rv2800, Rv2813, Rv2816 and Rv2825 genes and we found that the *truB* and *rbfA* genes are neighbours (data not shown).

Primers spanning the juncture of the orthologues of *Rv2800* and *Rv2825* in *M. smegmatis* and *M. avium* were designed (the upper primers hybridize to *Rv2800* and the lower primers to *Rv2825*) and used to confirm by PCR the *in silico* observation that these genes are neighbours in these species. The amplicons observed (Figure 2) confirmed the expected sizes; around 1 kb in *M. avium* and 0.55 kb *M. smegmatis*. As expected, amplification was not observed in *M. tuberculosis* because the hybridization sites of the primers are too far apart.

### Discussion

Despite the sequence conservation showed by most of the mycobacterial genomes so far sequenced, a genomic segment containing 21 genes and the DR region present in the *M. tuberculosis* complex is absent in *M. avium*, *M. smegmatis*, *M. marinum* and *M. leprae*. This region has probably been acquired by *M. tuberculosis*. This conclusion is based on the hypothesis that *M. avium* is older than *M. tuberculosis* (Sreevatsan *et al.*, 1997). *M. avium* and *M. smegmatis* probably appeared earlier in the evolution of this clade than *M. tuberculosis* because the genomes of these bacteria are longer than that of *M. tuberculosis*. *M. marinum* also seems to have a large genome (**http://www.sanger.ac.uk/Projects/M_marinum/**). Progressive deletions are proposed to have accumulated during the evolution of the *M. tuberculosis* complex, leading to speciation (Brosch

Copyright © 2004 John Wiley & Sons, Ltd. 

**Table 2.** Distances between ORFs that flank the DR region in *M. tuberculosis*

| | ***M. avium*** | | ***M. tuberculosis*** | | ***M. smegmatis*** | | ***M. leprae*** | | ***M. marinum*** | |
|---|---|---|---|---|---|---|---|---|---|---|
| **Gene** | **Position** | **Delta (kb)[a]** | **Position** | **Delta (kb)[a]** | **Position** | **Delta (kb)[a]** | **Position** | **Delta (kb)[a]** | **Position** | **Delta (kb)[a]** |
| Rv2793/truB | 3 810 970 | | 3 102 367 | | 155 877 | | 1 870 999 | | 405 450 | |
| Rv2800 | 3 813 339 | 2.4 | 3 108 416 | 6.0 | 159 842 | 4.0 | 1 874 326 | 3.3 | 400 324 | 5.1 |
| Rv2825 | 3 815 567 | 2.2 | 3 132 895 | 24.5 | 162 020 | 2.2 | 1 875 360 | 1.0 | 394 676 | 5.6 |
| Rv2831/echA16 | 3 815 922 | 0.35 | 3 135 791 | 2.9 | 162 357 | 0.33 | 1 876 260 | 0.9 | 392 937 | 1.0 |
| Rv2838/rbfA | 3 818 714 | 2.8 | 3 144 623 | 8.8 | 174 879 | 12.5 | Invert[b] | | 382 741 | 10.2 |
| Rv2845/proS | 3 825 596 | 6.8 | 3 151 205 | 6.5 | | | 1 877 242 | 1.0 | | |

[a] Distance with respect to the previous gene.
[b] Located downstream of *proS* in *M. leprae*.

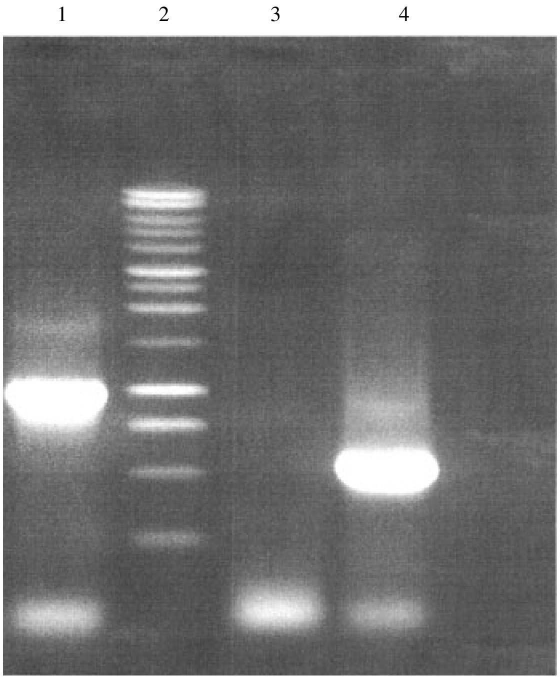

**Figure 2.** Amplification of the *Rv2800–Rv2825* region in *M. smegmatis* and *M. avium*. Lanes: 1, *M. avium*; 2, molecular weight markers (from bottom to top, 0.25, 0.5, 0.75, 1, 1.5, 2, 2.5, 3, 4, 5, 6, 7 and 8 kb); 3, negative control; 4, *M. smegmatis*

*et al.*, 1992); this is also the case for *M. leprae* (Eiglmeier *et al.*, 2001). In consequence, it is more likely that *M. tuberculosis* has gained a fragment than the occurrence of several independent events in which *M. avium*, *M. smegmati* s, *M. marinum* and *M. leprae* lost the same fragment.

The function of most of the proteins encoded by *Rv2801–Rv2824* is unknown, but several of the proteins encoded by ORFs upstream of the DR locus (*Rv2801*, *Rv2807*, *Rv2810*, *Rv2812*) are similar to DNA binding proteins or putative transposases. *Rv2805* and *Rv2807* seem to be duplicated genes. Downstream of the DR, no significant homologies were found. The expression of one of the genes, *Rv2813*, has previously been demonstrated by RT–PCR (Rindi *et al.*, 1999). It is difficult to draw conclusions from the GC% of this region, as it is slightly lower than the genome (63.46% vs. 65.6%). It appears that several of these genes are not required for virulence, as they are absent in several isolates associated with tuberculosis outbreaks: R*v2816–Rv2819* are missing from the genome of the Beijing strain 120 that was sequenced by TIGR. A strain that provoked a vast nosocomial TB outbreak in Argentina (Ritacco *et al.*, 1997; Alito *et al.*, 1999) also lacks this region (data not shown). R*v2816–Rv2818* were lost in some strains of the *M. microti* genome in a deletion termed MiD1 (Brodin *et al.*, 2002). In contrast, the *Rv2800* and *Rv2825* orthologues may be essential, because both genes are intact in *M. tuberculosis*, *M. bovis*, BCG, *M. avium* and *M. smegmatis*. Both of these genes are pseudogenes in *M. leprae*, but as it is an obligate parasite it is likely that it requires fewer genes to grow. Sequences with a CRISPR-type organization have been described in *M. avium*, but are located elsewhere in the genome (Jansen *et al.*, 2002).

The molecular mechanism that has led to the insertion of this fragment in *M. tuberculosis* is unknown, but it is possible to speculate that the putative transposases present in the upstream flank of the DR could have played a role in

Copyright © 2004 John Wiley & Sons, Ltd. 

a hypothetical insertion into the *M. tuberculosis* chromosome. Significantly, *Rv2813* is highly similar (expect score = e−117, 245/270 identical amino acids) to a gene found on an *M. celatum* plasmid, close to a double 18 bp tandem repeat (data not shown) with no function identified (Picardeau and Vincent, 1998; Picardeau *et al*., 2000; Le Dantec *et al*., 2001). All these features are reminiscent of the organization of the DR region in *M. tuberculosis* ($2 \times 18$ bp = 36 bp) and suggest that the DR region could have been acquired from a mycobacterial plasmid, not necessarily from pCLP1. Several authors have proposed horizontal transfer as an explanation for genetic exchange amongst mycobacteria (Howard *et al*, 2002; Le Dantec *et al*., 2001; Parsons *et al*., 1998) but mycobacterial horizontal transfer is a controversial subject.

In summary, we propose that the DR region was acquired by the *M. tuberculosis* complex or by an ancestral bacterium. The genomes of other mycobacterial species are currently being sequenced, and by extending our analysis to them, we should be able to confirm or discard this hypothesis.

### References

Alito A, Morcillo N, Scipioni S, *et al*. 1999. The IS*6110* restriction fragment length polymorphism in particular multidrug resistant *Mycobacterium tuberculosis* strains may evolve too fast for reliably use in outbreak investigation. *J Clin Microbiol* **37**: 788–791.

Brodin P, Eiglmeier K, Marmiesse M, *et al*. 2002. Bacterial artificial chromosome-based comparative genomic analysis identifies *Mycobacterium microti* as a natural ESAT-6 deletion mutant. *Infect Immun* **70**: 5568–5578.

Brosch R, Gordon SV, Marmiesse M, *et al*. 2002. A new evolutionary scenario for the *Mycobacterium tuberculosis* complex. *Proc Natl Acad Sci USA* **99**: 3684–3689.

Caimi K, Romano MI, Alito A, *et al*. 2001. Sequence analysis of the direct repeat region in *Mycobacterium bovis*. *J Clin Microbiol* **39**: 1067–1072.

Eiglmeier K, Parkhill J, Honore N, *et al*. 2001. The decaying genome of *Mycobacterium leprae*. *Lepr Rev* **72**: 387–398.

Fang Z, Morrison N, Watt B, Doig C, Forbes KJ. 1998. IS*6110* transposition and evolutionary scenario of the direct repeat locus in a group of closely related *Mycobacterium tuberculosis* strains. *J Bacteriol* **180**: 2102–2109.

Groenen PMA, Bunschoten AE, van Soolingen D, van Embden JDA. 1993. Nature of DNA polymorphism in the direct repeat cluster of *Mycobacterium tuberculosis*: application for strain differentiation by a novel typing method. *Mol Microbiol* **10**: 1057–1085.

Hermans PWM, van Soolingen D, Bik EM, *et al*. 1991. The insertion element IS987 from *Mycobacterium bovis* BCG is located in a hot spot integration region for insertion elements in *Mycobacterium tuberculosis* complex strains. *Infect Immun* **159**: 2695–2705.

Howard ST, Byrd TF, Lyons CR. 2002. A polymorphic region in *Mycobacterium abscessus* contains a novel insertion sequence element. *Microbiology* **148**: 2987–2996.

Jansen R, van Embden JDA, Gaastra W, Schouls LM. 2002. Identification of genes that are associated with DNA repeats in prokaryotes. *Mol Microbiol* **43**: 1565–1575.

Jansen R, van Embden JD, Gaastra W, Schouls LM. 2002. Identification of a novel family of sequence repeats among prokaryotes. *OMICS* **6**: 23–33.

Kamerbeek J, Schouls LM, Kolk A, *et al*. 1997. Simultaneous detection and strain differentiation of *Mycobacterium tuberculosis* for diagnosis and epidemiology. *J Clin Microbiol* **35**: 907–914.

Le Dantec C, Winter N, Gicquel B, Vincent V, Picardeau M. 2001. Genomic sequence and transcriptional analysis of a 23-kilobase mycobacterial linear plasmid: evidence for horizontal transfer and identification of plasmid maintenance systems. *J Bacteriol* **183**: 2157–2164.

Legrand E, Filliol I, Sola C, Rastogi N. 2001. Use of spoligotyping to study the evolution of the direct repeat locus by IS*6110* transposition in *Mycobacterium tuberculosis*. *J Clin Microbiol* **39**: 1595–1599.

Mojica FJM, Ferrer G, Juez G, Rodriguez-Valera F. 1995. Long stretches of short tandem repeats are present in the largest replicons of the archea *Haloferax mediterranei* and *Haloferax volcanii* and could be involved in replicon partitioning. *Mol Microbiol* **17**: 85–93.

Parsons LM, Jankowski CS, Derbyshire KM. 1998. Conjugal transfer of chromosomal DNA in *Mycobacterium smegmatis*. *Mol Microbiol* **28**: 571–582.

Picardeau M, Vincent V. 1998. Mycobacterial linear plasmids have an invertron-like structure related to other linear replicons in actinomycetes. *Microbiology* **144**: 1981–1988.

Picardeau M, Le Dantec C, Vincent V. 2000. Analysis of the internal replication region of a mycobacterial linear plasmid. *Microbiology* **146**: 305–313.

Rindi L, Lari N, Garzelli C. 1999. Search for genes potentially involved in *Mycobacterium tuberculosis* virulence by mRNA differential display. *Biochem Biophys Res Commun* **258**: 94–101.

Ritacco V, Di Lonardo M, Reniero A, *et al*. 1997. Nosocomial spread of human immunodeficiency virus-related multidrug-resistant tuberculosis in Buenos Aires. *J Infect Dis* **176**: 637–642.

Sola C, Devallois A, Horgen L, *et al*. 1999. Tuberculosis in the Caribbean: using spacer oligonucleotide typing to understand strain origin and transmission. *Emerg Infect Dis* **5**: 404–414.

Sreevatsan S, Pan X, Stockbauer KE, *et al*. 1997. Restricted structural gene polymorphism in the *Mycobacterium tuberculosis* complex indicates evolutionarily recent global dissemination. *Proc Natl Acad Sci USA* **94**: 9869–9874.

van der Zanden AGM, Kremer K, Schouls LM, *et al*. 2002. Improvement of differentiation and interpretability of spoligotyping for *Mycobacterium tuberculosis* complex isolates by the

Copyright © 2004 John Wiley & Sons, Ltd. 

introduction of new spacer oligonucleotides. *J Clin Microbiol* **40**: 4628–4639.

van Embden JD, van Gorkom T, Kremer K, *et al*. 2000. Genetic variation and evolutionary origin of the direct repeat locus of *Mycobacterium tuberculosis* complex bacteria. *J Bacteriol* **182**: 2393–2401.

van Soolingen D, de Haas P, Hermans PWM, Groenen P, van Embden JDA. 1993. Comparison of various repetitive DNA elements as genetic markers for strain differentiation and epidemiology of *M. tuberculosis*. *J Clin Microbiol* **31**: 1987–1995.

Copyright © 2004 John Wiley & Sons, Ltd. 
